# The relationship between speculation and translation in Bioethics: methods and methodologies

**DOI:** 10.1007/s40592-023-00181-z

**Published:** 2023-09-28

**Authors:** Tess Johnson, Elizabeth Chloe Romanis

**Affiliations:** 1 Ethox Centre, Nuffield Department of Population Health, University of Oxford, Oxford, UK; 2 Centre for Ethics and Law in the Life Sciences, Durham Law School, University of Durham, Durham, UK; 3 Petrie-Flom Center for Health Law Policy, Biotechnology and Bioethics at Harvard Law School, and Edmond and Lily Safra Center for Ethics, Harvard University, Cambridge, MA USA

**Keywords:** Speculative bioethics, Translational bioethics, Impact, Ectogestation, Enhancement

## Abstract

There are increasing pressures for bioethics to emphasise ‘translation’. Against this backdrop, we defend ‘speculative bioethics’. We explore speculation as an important tool and line of bioethical inquiry. Further, we examine the relationship between speculation and translational bioethics and posit that speculation can support translational work. First, speculative research might be conducted as ethical analysis of contemporary issues through a new lens, in which case it supports translational work. Second, speculation might be a first step prior to translational work on a topic. Finally, speculative bioethics might constitute different content altogether, without translational objectives. For each conception of speculative bioethics, important methodological aspects determine whether it constitutes good bioethics research. We conclude that whether speculative bioethics is compatible with translational bioethics—and to what extent—depends on whether it is being employed as tool or content. Applying standards of impact uniformly across bioethics may inappropriately limit speculative bioethics.

## Introduction

There is considerable and increasing pressure placed on bioethics researchers to demonstrate the impact of their research. The increasing emphasis on securing a concrete return on academic investments from policymakers has been a part of the neoliberal agenda in Europe and the US since the 1990s (Holbrook 2017). This political move aligns with shifts in some academic disciplines toward ‘translational’ work, which may be unsurprising given that in some cases, work that affects the economy, society, or policy is worth 6-7 times more to universities, in financial terms, than research outputs (Watermeyer and Chubb 2019). Similarly, funders increasingly expect researchers to build plans for impact and translation from the academic context to applied contexts into their applications for funding support. Bioethics is no exception.

Given that bioethics sits comfortably within applied ethics, it also has embedded within it an expectation that it has a practical orientation. Beyond the general expectation of practical outcomes in the policy and clinical or research practice, we have seen the emergence of the sub-field of ‘translational bioethics’ (Cribb 2010; Matthews et al. 2016; Sisk et al. 2020). For many, translational bioethics is simply bioethics: this is what ought to be defining the field. This has become something of a contentious point over the last few decades: there are questions asked about the degree of practical application that work must have “to have value or ‘count’ as bioethics. Whilst some recent work has emphasised the important place of philosophy in bioethics (Blumenthal-Barby et al. 2021), nonetheless it is often asserted that bioethics must be ‘action-guiding’ and ‘should tell us what to do’ (Chan 2015, 18). Translational bioethics highlights and explores existing ‘real-world bioethical issues’ and focuses on bridging the gap between academic reflection and current policy and practice. As Matthews et al. put it, translational “efforts are required to help us further mature and coalesce as a field of inquiry and will position bioethics as a leader in the conversation about assessing the impact of academic research and scholarship” (2016, 39). While we agree that translational work in bioethics (whether conceived as definitionally a part of the field, or as a sub-field) is important, we worry that this might draw emphasis away from valuable methods and content that currently features within bioethics as ‘speculation’. We note, also, that there are other forms of valuable bioethics that are neither ‘translational’ nor ‘speculative’—we do not suggest that these two categories are binary. We focus on speculative bioethics as this is an area of research that might stereotypically be perceived as opposite to translational research. Moreover, we suggest that speculative methods and content and translational bioethics as a sub-field are not necessarily mutually exclusive, in that speculative methods may be useful for work in translational bioethics, but also, we defend speculative content in bioethics that has no imminent real-world practical applications.

Speculative bioethics is often thought of exclusively as the discussion of future technologies (Schick 2016). As Racine et al. conceive it, speculation is “an effort to foresee potential or probable scenarios and their outcomes based on assumptions that cannot be verified by empirical or scientific claims in the present” (2014, 326). Like Racine et al., we take speculative bioethics to include a range of forms of inquiry, all related by being—at their core—a form of practical/applied ethics in relation to less temporally immediate problems from a range of contextual possibilities not limited to, but including, imaginaries, thought experiments, and future technologies. McMillan explains that there are “six distinct forms of speculative reason that are used in bioethics. These are: speculative practical reason, the counterexample, the argument by analogy, an attempt to deepen moral understanding, the intuition pump, and the heuristic device” (2008, 128). All these forms of reasoning, as practical ethics without a clear application to an immediate bioethical issue, at first glance appear oppositional to translational bioethics; they do not appear to have the immediate objective of changing current policy or practice.

In this paper, we argue that speculative bioethics methods and content have several important functions for the field. We explore speculative ethics both as method and content, and in examining each form we draw conclusions on the relationship between speculative bioethics and standards that might feature in translational bioethics. First, we consider speculation as a method, a means of reflecting on contemporary existing issues. Second, we consider speculation again as a method, as a potential precursor to translational work. In relation to these two purposes, then, our argument is that speculative bioethics—whatever method deployed—has a clear utility in *enabling* translational work. Third, we consider speculative bioethics as a unique line of inquiry into content with limited connection to translational or impact objectives. Despite there being no translational quality to this work, we argue that it has clear justification and purpose.

As a caveat to our defense of speculative bioethics, we note that critical introspection on the part of scholars undertaking speculative work is key. When setting up thought experiments about novel technologies or setting out the prospect of a future technology for examination, scholars must be clear about where the value comes from in the speculation they undertake. We do not think that in defending speculative bioethics, we must defend instances of the methods not being done reasonably well. For this reason, we highlight the importance of researcher reflexivity, positionality, and the avoidance of technological determinism. We take these to be fundamental methodological components to good speculative scholarship that can ensure speculative work has the benefits that we outline. When these conditions are met, speculative bioethical work constitutes a valuable set of methods and line of independent bioethical inquiry.

## Speculation as a theoretical lens

Speculation affords researchers room for imagination. There are several ways in which imaginative ideas and scenarios can bring life to an argument, and others in which they create space for novel thinking and argumentation. In this way, speculative bioethics is a tool that, through the use of sometimes fantastical or futuristic scenarios, can allow for better investigation of practical ought questions. Moreover, as we also illustrate in this section, thinking about novel technologies—that do not yet exist—in a contemporary context can also contribute to increasing the visibility of contemporary problems. In this section, we explore speculation as a theoretical lens in bioethics: first, in that imagining various scenarios or thought experiments can force us to elucidate our intuitions and test guiding principles, and second, in that they can draw further attention to problems in context. In this way, speculative bioethics can act as another means of performing translational bioethics.

### Method 1: testing intuitions and principles

Thought experiments, for example, are an elegant way of constructing a scenario that can center or highlight a particular ethical problem in its purest sense. Thought experiments can be a form of both fiction and of speculative thinking and are important in giving us “a starting point to engage with the issues as well as to develop and refine our analyses of the problem; they present the ethical arguments in an immediately accessible form and inspire debate” (Chan 2009, 398). Taking imagined scenarios and using them to ask questions is not necessarily intended to represent actual scenarios that do or could occur in real life, but rather to act as “operations providing premises for arguments” (Häggqvist 1996, 136). The researcher does not always set out to *solve* the imagined problem in a way that is determinative or even to say what ought to be done if this matter were ever to materialise, but rather “to provide some sort of structure to how we think about the much more complex, messy, squishy problems we encounter in real life—to abstract certain dimensions of these problems and render them susceptible of analysis” (Chan 2015, 18). Thought experiments can, therefore, help us concentrate on a problem (or show clearly why something is not the problem it is presented as) by distilling it.

As an example, Rachels, in his book about the morality of euthanasia, seeks to demonstrate the ‘equivalence thesis’ that “there is no morally important difference between killing and letting die” such that if it is permissible for doctors not to provide treatment, it must also be permissible for them to hasten a death (Rachels 1986, 111). To investigate the truth of this specific point, Rachels introduces a thought experiment in which two men—Smith and Jones—each stand to gain financially from the death of their six-year-old cousins. Smith drowns his cousin in the bath and makes it look like an accident. Jones plans to drown his cousin, however, on entering the bathroom his cousin slips, hits his head and falls unconscious into the bathwater. Jones does not help, and his cousin drowns (Rachels 1986, 113). Rachels explains that “if the difference between killing and letting die were itself a morally important matter, then we should say that Jones’s behaviour was less reprehensible than Smith’s” (1986, 113). He uses this as the starting point for his equivalence thesis, arguing that the men are equally morally reprehensible because their motives and the results of their conduct are the same. By creating this scenario, Rachels can focus on the key question at hand in the purest sense. The example is also able to challenge the reader’s intuitions about what it is that morally matters by developing an imagined scenario to then make moral inferences about euthanasia.

While this distillation of complex issues to a thought experiment with a clear problem in need of a solution can be useful in assisting as we begin to grapple with a novel context or problem (or even an old one), it might also be criticised for its over-simplification of the “more complex, messy, squishy problems” (as Chan put it) in real life. For example, the Smith and Jones thought experiment might be criticised for oversimplifying the issues involved in euthanasia because it takes out contextual factors like doctors’ motivations, a doctor’s broader obligations, the patient’s motivations, the specific mechanics of the doctor-patient relationship, and the social status of doctors, just to name a few. It also assumes that judgements about equivalent impermissibility can be used to make inferences about the equivalence of permissible cases. We agree that contextual factors matter. We posit, however, that a thought experiment can also do a serious amount of heavy lifting in illustrating the importance of context. First, sometimes the facts can be tweaked slightly, and this changes the conclusions reached—which, in effect, performs the useful task of illustrating that a particular contextual variable really matters. Second, well-argued work spends a significant amount of time explaining why the thought experiment has utility in elucidating a principle that can work in the specific translational context.

Furthermore, thought experiments can illuminate some of the assumptions that underlie our intuitions and ideas by stretching the circumstances in which we apply them. The utility here (and this also explains why thought experiments are so useful at teasing out new ideas or encouraging students to think through ideas) is that they challenge us to explain and justify argumentation to a greater degree. As Walsh explains, this need not always be a situation that is empirically possible: “if an argument regarding such a case relies centrally on a general moral principle, then it is warranted to test its generality against a range of possible scenarios, which may or may not be modally bizarre” (2011, 474). This can enhance our conceptual understanding, even if our contextual understanding is influenced by other things. All of this work should be thought of as a potential precursor to translational work—indeed, we may well *need* to understand moral principles in theory before we begin applying them, as Blumenthal-Barby et al. note when emphasising the important role of philosophy in bioethical work (2021).

### Method 2: enhancing the visibility of contemporary problems

Speculation often has the capacity to enhance the visibility of what should be considered contemporary bioethical problems—particularly structural inequality. As Chan explains, “[t]he value of conducting thought experiments that are themselves unrealistic and speculating about technological developments which are as yet only futuristic possibilities lies not least (though also, I would venture to suggest, not only) in what such considerations can tell us about real and present bioethical problems” (2015, 18). There are lots of examples in recent literature of scholars imagining future and novel technologies in contemporary contexts. There is a growing body of feminist bioethics literature exploring what artificial placenta technologies/ectogestation might mean for reproductive autonomy (Cavaliere 2020a; Horn 2022; Horn and Romanis 2020; Kendal 2015; Nelson 2022; Romanis et al. 2021) and sex/gender equality (Cavaliere 2020b; Hooton and Romanis 2022; MacKay 2020). Much of this literature is clear (importantly) that these investigations are speculative because the technology, in its current iteration, is not capable of replacing pregnancy (Horn and Romanis 2020). There is, similarly, a growing body of work on human enhancement that uses futuristic examples to highlight current problems with more everyday issues of biomedical enhancement, reproductive autonomy, and health justice (Anomaly 2020; Bostrom 2003; Gyngell and Douglas 2015; Johnson 2021a 2021b; Persson and Savulescu 2012).

Imagining farfetched technological advances in the present might be criticised as lacking utility because social circumstances would likely have evolved before such possibilities come to fruition (maybe 100 years into the future). However, this criticism is reliant on the utility of the investigation only being what we ought to do *in the advent of the technology*. We explore the importance of such enquiries later in this paper. Here, however, we defend these investigations because of what they can tell us about problems in the present: principally, imagining how novel technologies might be used in the present is able to magnify contemporary issues—particularly problems in how people are treated and the scale of inequality (and in the next section, we investigate how explorations of this speculative content can themselves constitute valuable bioethics, too, even ignoring the usefulness as a tool). Looking back to the example of artificial placenta technology, in imagining how artificial placentas will impact on pregnant people, authors have been able to illustrate how phenomena that underlie contemporary obstetric/pregnancy care provision are dangerous.

The ‘maternal-fetal conflict’ framing of pregnancy perpetuates the notion that the interests of pregnant people and fetuses are, or can be, in direct conflict (Baylis et al. 2008). Romanis et al. (2021) use the example of artificial placenta technologies to exemplify further why we need a wholescale shift away from this conceptualisation of pregnancy. While there has been exploration of the conceptual and practical problems with ‘maternal-fetal conflict’ (Bowden 2019), imagining the use of the technology in contemporary context magnifies the current problem. It shows the extremities of how current modes of practice are dangerous by showing the outcomes when enabled by technology are dangerous. Romanis et al. reflect on how artificial placenta technologies could be used as a tool to further coerce pregnant people’s behavior. They use the artificial placenta to bolster their conclusion that it is imperative that we begin “[r]eorienting our understanding of pregnancy away from maternal–fetal conflict” (2021, 829). Similarly, Horn (2022) uses the imagining of the artificial placenta in contemporary context to illustrate the limitations of reproductive autonomy and freedom in ensuring access to novel technologies. There is a considerable body of work that critiques reproductive health inequalities along the strata of race and class—specifically reflecting on the ways in which technologies heralded as tools of liberation for some become tools of oppression for marginalised groups (Roberts 2017). Horn’s work meaningfully adds to this by producing powerful imagery. Considering the artificial placenta within the contemporary (longstanding) inequalities and systematic reproductive racism in pregnancy care, Horn shows us a world in which white women have a choice about what risks they want to assume in becoming a biological parent, and black women are coerced into *ex utero* gestation. This is another way of illustrating the scale of contemporary reproductive injustice. Finally, several authors have used the artificial placenta to show the problems with the ways in which abortion rights are constructed in several jurisdictions, making them vulnerable (Horn 2020; Romanis 2020).

In the genetic enhancement context, scholars using futuristic examples are doing something similar. By discussing what a genetically enhanced posthuman future might look like (Anomaly 2020) we can highlight existing issues with access to the very fertility services (like in vitro fertilisation) that are necessary for genetic enhancement to begin with (Gyngell and Douglas 2015). If genetic enhancement were implemented today, without further healthcare capacity-building around the world to satisfy requirements of global justice, without equalszing access to healthcare within countries, and without a more collectivist perspective on enhancement in general to address collective action problems, the posthuman future that we might envision in the thought experiment—regardless of whether it is good or bad for the enhanced—would only apply to the privileged few with access to advanced fertility services (Johnson 2021a). Similarly, we might use scenarios involving genetic selection technologies, sometimes discussed as a predecessor of genetic enhancement, to discuss issues of reproductive autonomy more broadly (Johnson 2021b).

What unifies all these examples is that they use a technology to magnify an existing problem: imagining novel technologies into contemporary context can magnify existing inequalities or the problems in existing frameworks for decision-making. As Sheehan and Dunn note “[t]he fact that theoretical abstractions are used to make practical ethical arguments need not make the resulting claims any less practical than would be the case in the application of social theoretical insights to the interpretation of empirical facts about social processes in the world” (2013, 56).

## Speculation as a precursor to translational work

In addition to speculative bioethics acting as a tool in terms of a theoretical lens through which bioethicists can (by proxy) analyse contemporary issues, it can act as a tool in terms of a precursor to more translational work. Speculative bioethics can sometimes be a form of “proactive ethics” (Racine et al. 2014, 326). In this section, we propose two ways in which speculative bioethics prepares the ground for translational work to ensue once a futuristic technology or issue becomes imminent. The first concerns the identification of new research questions and areas of enquiry. The second concerns foundational work on necessary concepts or removing hindrances to future work.

### Method 1: identifying research questions

Speculative bioethics is useful as a precursor to later translational work as inspiration, a means of identifying new research questions and directions of enquiry. Consider the following thought experiment:

Suppose there were an experience machine that would give you any experience that you desired. Superduper neuropsychologists could stimulate your brain so that you would think and feel you were writing a great novel, or making a friend, or reading an interesting book. All the time you would be floating in a tank, with electrodes attached to your brain. Should you plug into this machine for life, preprogramming your life’s experiences? (Nozick 1974, 48)

Nozick aims to test our intuitions regarding whether what makes a life go well is the mental state of pleasure, or the satisfaction of desires or preferences. For those who would plug themselves into the experience machine, what matters seems to be the experience of pleasure, regardless of whether it comes from a false source or does not result from actually satisfying any desires. For those who would not plug in, what matters seems to be the satisfaction of preferences in the real world, authentic experience, or the source of pleasure rather than its mental consequences.

Nozick uses the thought experiment identify this valuable line of enquiry into what makes a life go well. The experience machine is his inspiration for developing the research question. Nozick engages in speculative bioethics—perhaps more as a side-effect of his more theoretical undertaking—by proposing the existence of such a machine. If we were faced with this choice, what should we do? It is a question not only for wellbeing theorists, but for future-thinking bioethicists. We might even say that Nozick’s speculative bioethics is our translational bioethics today. We are faced with decisions about how much time to spend online or in person that might be considered the modern equivalent to plugging into the experience machine. Men who played games in the US in 2019 spent an average of 2.88 hours per day doing so, whilst women spent 1.51 hours (Lock 2022). Do their lives go better, or worse, as a result of this online engagement? More broadly, recent research has shown that internet users aged 16–64 spent 2.27 hours per day on social media in 2021 (Kemp 2022). Do their online interactions with friends matter as much as their in-person ones? With increasing immersion in an online world, we are closer to the issues raised by the experience machine thought experiment. It was this then-speculative bioethics that paved the way for important contemporary bioethics research questions about whether we should spend our time online to make our lives go well.

Nozick is not the only academic to use speculative bioethics as a tool like this. Another example is Turing’s presentation of the ‘Artificial brain,’ which examines whether it is possible to create a machine with the same capabilities as a human and presents the Turing test as a means for determining whether a machine does display intelligent behavior indistinguishable from a human’s (Turing 1950). Turing introduced the thought experiment long before artificial neural networks began to be used, which aim to model artificial intelligence (AI) on learning mechanisms in the human brain, but the thought experiment raised questions that got bioethicists designing research questions about ethical issues of relevance to neural networks (Beavers 2011; Sparrow 2004) before the Turing test was first (according to some standards) passed, in 2014 (Sample and Hern 2014). In another instance, ‘Roko’s basilisk’ is a speculative case where a future artificial general intelligence might be incentivised to torture all people who knew of its development but failed to contribute to its existence (Less Wrong 2010). It has been used to discuss implicit religious commitments in ethics (Singler 2018) and informational hazards (Auerbach 2014), foundational issues producing research questions that might prepare the ground for later translational work on AI ethics. It may yet pave the way for further bioethical inquiry concerning the AI singularity before this becomes imminent. Speculative bioethics might be perceived as a good way to inspire new lines of thinking because it provides a space for creative thought without the parameters and practical boundaries that are more intuitively applied to every-day scenarios. Whilst there are limits to our abilities to make other animals’ brains equivalent to human brains through training, and perhaps ethical issues to boot, there is nothing to say an AI could not, theoretically, be made to exactly replicate a human brain, and indeed we are already part of the way there. Speculative bioethics initially plays the role of a thought experiment, and the work on that thought experiment can later be used to answer ethical questions about the technology/situation, should it eventuate.

### Method 2: pre-empting foundational issues and hindrances to translational work

Speculating can also allow us to stay ahead of developments, and is a means of ‘thrashing out’ foundational issues before the work becomes urgent. We note the possible objection to this line of thinking that it is a potential form of technological determinism—but we will address this in the final section about methodological caveats.

Take the ‘Moral machine’ platform, which engages the public in a set of online speculative scenarios concerning autonomous vehicles, online:

When it becomes possible to program decision-making based on moral principles into machines, will self-interest or the public good predominate? […] Autonomous vehicles (AVs) should reduce traffic accidents, but they will sometimes have to choose between two evils, such as running over pedestrians or sacrificing themselves and their passenger to save the pedestrians. Defining the algorithms that will help AVs make these moral decisions is a formidable challenge. (Bonnefon et al. 2016, 1573)

Moral machine helps to overcome this formidable challenge through early engagement before autonomous vehicles are (much) on the road. Participants are faced with moral dilemmas an autonomous vehicle may face and are asked what it should do. This work merges moral psychology, empirical bioethics, and speculative bioethics. It discovers people’s moral intuitions surrounding given scenarios and can use this empirical work to inform the issue of how to ethically program self-driving cars. It sets moral standards and works to answer questions such as, How do we value the life of a toddler in comparison to that of an elderly person? Should a passenger of a car be prepared to risk their life (and be considered to have implicitly consented to this) more than pedestrians walking across the street, if a car must decide between likely killing one or the other? Ethicists are using platforms like the moral machine to stimulate debate on these topics (Awad et al. 2018; Harris 2020). Whilst there are some self-driving cars on the road now, legal cases to date have mostly focused on drivers’ responsibility, given the limits of most autonomous systems to cruising conditions or use only when the driver’s hands are still on the steering wheel (Ipro Tech 2019). From the point of view of the law these harmful actions are still caused by humans. However, this might not be the case, quite soon. Tesla has already announced its intentions to have driverless ‘robotaxis’ on the roads by 2024 (Lambert 2022). Before such technologies are released, important ethical work must be done. The example of driverless cars shows this need in the short-term future, and the need for more empirical work to determine how autonomous vehicles should be programmed to respond to moral dilemmas in potential crash scenarios.

The same goes for longer-term ethical issues, however. Foundational work that may be necessary before the technology is imminent, is no less important for more futuristic speculative bioethical issues. Much inquiry recently has been dedicated to the question of how we should prioritise our research and development efforts. Setting global priorities for scientists, economists, and ethicists is the goal of whole research institutes (Global Priorities Institute 2022) some of which have significant influence on funding and research decisions across institutions and countries. If practical ethicists should spend their time effectively on solving the most important ethical issues, then speculative bioethics contributes a tool for achieving this goal. Most of the people who will ever live are yet to be born and will be born into a world very different from the current one. Speculative bioethics deals with issues that may be more relevant to the lives of many future people who may live in worlds with artificial placentas, genetically enhanced people, and autonomous vehicles. Some philosophers who work on global priorities argue that much more work is needed—in ethics and other areas—that looks to the longer term (Ord 2020). In doing speculative bioethics work, we ensure that some of today’s ethical issues that might be redundant in the future do not monopolise all our attention.

## Speculative bioethics is valuable

In this section, we present speculative bioethics not as a tool that might or might not be used in bioethics with translational goals, but as separate content, as its own valuable line of bioethical inquiry.

Much work has gone into defining bioethics and outlining its remit. And yet, disagreement remains (McMillan 2008). We might first consider whether there is a distinction between bioethics and applied ethics. Whilst philosophers view applied ethics as their own remit, as the domain of philosophers, this is not the case for bioethics, which has involved lawyers, doctors, philosophers, sociologists, and others from its inception (McMillan 2008). This distinction helps us to understand, also, how speculative content in bioethics is not merely a move along a practical-theoretical spectrum from bioethics at the more practical end, to applied ethics with its more philosophical roots. A focus on more futuristic developments does not necessarily imply a change in methodologies or core research questions—it does not demand armchair philosophy instead of empirical work—and it does not disqualify scholars from diverse areas from investigating ethical questions relating to the speculative scenarios. Rather, speculative bioethics is still a form of bioethics—a term that itself requires further definition. Whilst some view bioethics as a field of inquiry, others view it as a discipline or as a governance practice (McMillan 2008). Whilst some define it in terms of a set of primary and secondary questions (Sheehan and Dunn 2013), others stipulate the appropriate methodologies that it uses (Sulmasy and Sugarman 2001). As Blumenthal-Barby et al. (2021) have recently observed, defining the field comes with assumptions, too, about the correct role of more philosophical, more normative and foundational work that might support other lines of bioethical inquiry. These discussions are relevant to our work here insofar as speculative bioethics tools and content are useful in the overall research area, either as a direct part of bioethics, or as part of the philosophical foundations that ground good bioethical work.

As Sheehan and Dunn point out, bioethics is about what ought to be done in the context of human interactions with the world (2013). It is a common tenet that you cannot have an obligation to do what you are unable to do, and so we might think that bioethics can only examine topics where there are current actions available to agents. That might not hold for many topics in speculative bioethics, where a technology has yet to be developed or an issue has yet to unfold. But, as Sheehan and Dunn note, such future issues, insofar as they can be connected to a specific context where there is a practical ought question involved, might still constitute bioethics, as long as this speculative content is “practical in an immediate sense” (2013, 57) where immediacy is interpreted, not temporally, but in terms of being pressing, important or urgent. They explain:

[B]eing practical is not just about being relevant to action, or to a consideration that may have some partial role in action. Nor is it enough simply to orientate an argument and a related claim towards a specific domain of practice, or to articulate a range of different perspectives that might influence obligations in “bio” contexts. Rather, the arguments to guide action in the specific context must function to actually guide action in that context: an answer to a practical “ought” question must provide direct, prescriptive guidance to agents in the relevant context such that these agents can be convinced to act by the claim being made. (Sheehan and Dunn 2013, 58)

If this is right, speculative bioethics can serve to provide secondary questions that must be answered as a means to more translational work, or as a lens that clarifies primary questions through situating them in a different context, or, in limited cases, as a primary question, an independent set of content for bioethical inquiry. The limitations of this lie with the requirement for the issue to be pressing and for it to be possible to specify a particular context in which a practical ought question is situated. This would exclude, for example, speculative content that is extremely abstract, or does not contain a practical ought question (for instance, some discussions of the ubiquitous trolley problems). Whilst we challenge Sheehan and Dunn on the vague aspects of this definition, it cannot be faulted for mistakenly excluding bioethics with speculative content.

Sheehan and Dunn (2013) avoid using a temporal interpretation of the term ‘immediate’ because it would exclude valuable bioethical discussions of, say, whether euthanasia is appropriately performed by doctors in countries where it is currently prohibited. It would, furthermore, exclude judgements of whether and how we should render future people genetically immune to certain diseases, upon the supposition that this is too far in the future—despite having already been attempted via genome editing (Johnson and Giubilini 2021). It would exclude devil’s-advocate bioethical arguments that aim to explore the logical conclusions of positions we already take, as in work conducted on analogies between abortion and infanticide (Giubilini and Minerva 2013). Blumenthal-Barby et al. might define these lines of inquiry as part of the philosophical work that has a legitimate role in bioethics (2021). Indeed, they use a very similar example to some of those above in their work:

A second way in which philosophy continues to be important in contemporary bioethics is in the context of new and emerging issues where philosophy is especially relevant to help clarify and address them. Consider, for example, the recent development of brain (cerebral) organoids for research purposes. These are artificially grown miniature organs resembling the brain, created using pluripotent stem cells. Are brain organoids conscious? How do we know? Should we treat them as if they are conscious? Answering these pressing bioethical issues involves delving into philosophy of mind and philosophical views on the nature and value of consciousness. (Blumenthal-Barby et al. 2021, 3)

The risk of this high level of inclusivity is that it may wrongly include parts of ethics, metaethics and philosophy of mind, among other areas, that are relevant to bioethics, but not linked enough to be considered a part of it. Indeed, Sheehan and Dunn’s definition of bioethics might exclude parts of the example above, both for not containing a practical ought question throughout (e.g., in the question of ‘are brain organoids conscious’) and for lacking context specificity. We might intuitively prefer, then, a slightly less inclusive idea of bioethics that still leaves room for some speculative content. However, in not requiring temporal immediacy, yet maintaining an emphasis on context-specificity, Sheehan and Dunn’s (2013) definition of bioethics seems too vague to clearly include or exclude much speculative content. If we can consider that future problems (that involve a practical ought question) are pressing and will even be immediate in a temporal sense, *eventually*, then for the purposes of determining whether speculative content is part of bioethics, how might we define ‘eventually’? A balance must be struck, because whilst immediacy may not be time-sensitive in the way they conceive it, context-specificity may plausibly decline as we explore more far-fetched, futuristic content. At what point can we no longer provide enough (plausible) context-specificity for speculative content to be considered part of bioethics? For that matter, must the actions that bioethics examines be *certainly* available at any point at all? Surely contingent evaluations that lack some context-specificity and rely on uncertain but plausible premises are also valuable. Say, if a conservative government is elected, then the actions available to doctors regarding the provision of abortion might be limited, and doctors may have to choose between referring pregnant people to clinics across states or leave them without provision. But then if a liberal government is elected, the actions available to doctors might expand, and they may not have the option to leave the patients without access to abortion, but rather to always offer abortion. The options available to doctors depend on which government is elected. No set of actions is certainly available in the future. Should bioethicists not bother investigating what doctors should do in each case? Surely not. Rather, we might expect bioethical inquiry to provide doctors with contingent courses of action for the possible eventualities and possible relatively specific contexts they might encounter in future. This kind of exploration of contingent questions and situations is ‘speculative’ in a way that still seems practical and important for bioethics.

We have argued some definitions of bioethics may be too vague to clearly include or exclude speculative content. And yet it seems there is a clear case for including speculative content, regardless of current definitions. We might instead think that bioethics should prepare its targets for plausible future eventualities. In that case, there may be room for speculative bioethics independent of translation. We might think that speculative bioethics can prepare us for the development of technologies that may never occur, but concerning which we would need ethical guidance, if they ever do become available. This may be what Cribb intends to allow, when he claims: “Of course, […] translation is not everything and that we can imagine important contributions being made to our understanding of ethics where the value of these contributions is, in key respects, independent of what happens in practice” (2010, 208).

In that case, even separating out the value of speculation as a tool from speculative, futuristic content, it seems there might be a legitimate place for speculative content in bioethics. Problems that seem less than immediate or pressing, and where we cannot supply the specifics of context may still be worth investigating if there is a practical ought question in there somewhere. For instance, ought we embrace a posthuman future, involving radical changes to our physical and mental features? If so, how would we regulate use of the technologies that achieve this? The only requirement we might have of such speculative bioethics content is that it does not turn into pure abstraction, which might be valuable still, but would constitute theoretical ethics rather than bioethics. This might occur, for instance, by reducing the above line of enquiry to ‘would a posthuman future be a good thing?’

## Methodological reflections

While we have advocated very strongly for the value of speculative bioethics, both as tool and content, we want to reflect on what we see as standards of good practice. There is utility in future-orientated thinking (basing reasoning on complete unknowns or imaginaries) and anticipatory ethics (making predictions based on incomplete scientific evidence) (Racine et al. 2014). However, we believe that speculative bioethics, particularly when used as a tool, has the *most utility* where it is grounded in reflexivity. Grounded speculation encompasses engaging in anticipatory reasoning but with constraints clearly marked out by reflexivity on the part of the researcher as well as a solid scientific knowledge base (Racine et al. 2014). We argue that a ‘grounded’ approach to speculation enhances the rigour of the exercise, and that reflexivity is an important part of this approach—this is the case whether the speculative methods engaged are related to translation or not.

In this section, we address two important methodological constraints. First, the importance of acknowledging that work *is* speculative to avoid problems of technological determinism. Second, the importance of acknowledging researcher positionality and what values are taken as a given when deploying speculative method. We stress that this reflexivity is important for good speculative bioethics because researchers “must be able to critically assess visions of technological futures if it is to function as an ethics that is of and for the present” (Schick 2016, 226).

While speculation is important in bioethics, so is acknowledging that work *is* speculative, what it is speculating about, and why. Bioethicists do not tend to issue careful disclaimers about when they are overstating technological possibilities and this is often because, in the pressure to publish, one needs to assert that the ethical issue they are addressing is a pressing—and perhaps, exciting—one (Hedgecoe 2010; Romanis and Horn 2020). However, methodological rigour in speculation would be enhanced by some acknowledgment of what is and is not possible, and what may or may not be possible in the future, and why. For example, there is considerable debate in the literature about the possibility of enhancing polygenic traits. This is currently a scientific impossibility because the available gene editing tools are unable to successfully edit more than one gene at a time (Janssens 2015; Jostins and Barrett 2011)—although, this may soon be possible (Carlson-Stevermer et al. 2020). There are several scholars, however, who exercise responsibility in explaining why they are engaging with technology that is not readily available (Johnson and Giubilini 2021; Persson and Savulescu 2019). For instance, we might want to consider how two polygenic traits, altruism and intelligence, if they were prevalent in the population at higher levels, might result in utopic or dystopic scenarios, as a reason to consider *whether* research on polygenic editing should continue, and if so, *which* polygenic traits must be enhanced first to avoid dystopic scenarios—in this case, we might feel that widespread altruism enhancement is needed before we allow intelligence enhancements. The reasons for engaging with current technological impossibilities might also include becoming prepared for how technologies might co-evolve in the future (where the abilities we have with one technology might be improved by the advent of another). Our point here is simply acknowledging that disclaimers that accompany examples like this better ensure methodological rigour when using speculative bioethics as a tool.

Some scholars have argued that we need to be clear about speculation and its value because otherwise we fall into the trap of technological determinism; of failing to discuss whether we ought to develop a technology in the first place. There is an easy tendency in speculation for researchers to go past what technologies should we develop and why, to what ethical problems will we face when novel technologies are here—and this assumes we ought to welcome their development. As the example above shows, sometimes discussion of technological impossibilities or technologies that we perhaps ought not develop is necessary to determine what should be done in the first place, as with discussions of some infeasible genomic enhancements. The risk of harms from technological determinism arises when discussions are accompanied by a sense of inevitability, or when, even more obviously, the inevitability of a technological development features as a part of the argument for that very development. Such arguments are, in a way, begging the question, as demonstrated by Lewens’ argument for (epi)genetic enhancement: “any rigid stance against intervention in the processes of inheritance is absurd, because such interventions are inevitable and pervasive” (2020, 14). Whilst we might accept it is more difficult to formulate policy in line with an ethical stance against a technological intervention if it is likely to be widely available and easily accessible, this is not a reason against such an ethical stance to begin with. As Racine et al. note, in such cases, “bioethical discussions could have implicitly contributed to the argument that it is socially acceptable because it is inevitable. Such an argument clearly overextends what is known about non-prescription stimulant use with respect to its moral acceptability, praiseworthiness, and social desirability” (2014, 330).

Technological determinism also appears in arguments *against* certain technologies. Some scholars use slippery slope arguments to argue that *if* a certain initial technological development occurs, *then* it will inevitably lead to a further technological development or societal consequence that is obviously unacceptable. The mechanism of such a slippery slope is highly reliant on specific scientific advances, however, which is often neglected in the argument. For instance, both Sparrow (2016) and Mehlman (2012) argue against genomic enhancement due to developments of the technology and societal attitudes toward it that they assume are inevitable. Mehlman assumes that genomic enhancement would be both sufficiently powerful (implying polygenic editing is possible) and available via the private market, such that a genetic underclass would quickly emerge, consisting of those unable to afford enhancement (2012). Sparrow, similarly, argues against genomic enhancement on the basis of an inevitable slippery slope, claiming that “as it becomes more powerful, genetic enhancement will greatly increase the extent to which it is possible to engineer human beings for the benefit of the world, nation, or species. […] once one admits any role for the interests of third parties, this opens up the possibility that these interests might be very significant.” (2016, 138).

We are not arguing that researchers should limit themselves only to questions of whether we should develop a technology. Indeed, many examples of the utility of speculation that we have given in this paper speak directly to researchers going beyond these questions to what we ought to do if this technology does exist. Our reflection here is that to ensure that work is not determinist in nature, a small account of how the researcher sees their work in relation to the question of whether the technology ought to come into existence would be useful before the extension of slippery slope arguments or question-begging inevitability arguments.

Moreover, researchers must be reflexive about what values they take ‘as given’ when they construct speculative scenarios. When devising a thought experiment, or when undertaking an investigation of an emerging/future technology, researchers are relying on both empirical and normative premises. As Racine et al. explain, the specific act of speculation “takes place in the present, which… necessarily implies looking out from a particular place, or a particular point of view, towards an unknown future and thus, there are multiple ways to ‘look out’” (2014, 326). For this reason, researchers should explain what they have taken to be material in their approach to a novel technology, both for transparency, and to acknowledge limits in where this work has utility (e.g., if the technology can do X). At a more fundamental level, we suggest that researchers need to be clearer about what they are identifying as a problem and why.

A pertinent example comes from the literature surrounding artificial placenta technologies. Much of this has focused on the ethics of abortion and whether technology that can gestate outside the body enables a compromise: people can end pregnancies, but no fetuses die (Simkulet 2020; Singer and Wells 1985). Romanis and Horn (2020) have criticised the premises of this body of work; they argue that in the claim that artificial placentas are a compromise, the work is beginning with the premise that abortion is a ‘problem to be solved’. Yet, most scholars making such arguments do not acknowledge that this is an assumed premise in their work, nor do they offer a justification for this as their starting point, as opposed to, for example, the alternative starting point of abortion as healthcare. We argue that good speculative scholarship should be more forthcoming in the values underlying the investigation and the normative assumptions adopted as premises.

Bioethics could learn much from the practice in qualitative research wherein the researcher is encouraged to see themselves within their work and to think critically about what it is they have inserted of themselves into their generation of and interpretation of data (Bourke 2014). There is a much greater awareness that the researcher themselves features as a part of the process of generating data, measuring it, and using it (Braun and Clarke 2019, 2021). We imagine that if we asked the same questions of ourselves when speculating and constructing speculative work, and normalised publishing small accounts of how we came to the research and who we are in relation to the questions, this would increase the rigour of speculative work in encouraging us to be more self-aware and forthcoming in what we have taken for granted in our understanding of the topic or context.

## Conclusion

Speculative bioethics and translational bioethics are not dimorphous. Translational bioethics is a proposed sub-field or aspect of bioethics as a field that might usefully use speculation as a tool. Speculative bioethical content can form an independent line of bioethical inquiry too. There is a general trend towards requiring impact from bioethics work, with pressure coming from both inside and outside the field, however, which may emphasise translation in a way that excludes the valuable use of speculation in bioethics, and draws attention away from speculative content in the field. In this paper, we have argued that there are some ways in which speculative work has clear links to translational work. Speculative work can be important in raising public awareness and excitement about novel technologies, or even about the field of bioethics in general. It can be important in informing policymaking in matters only slightly related to the actual speculative work. What’s more, speculation is crucial in preparing the field and the public for involvement in translational discussions of emerging technologies in the future. The law and public policy are always struggling to keep up with scientific development (often because other matters are considered more urgent until the development itself becomes the urgent matter). One of the great advantages of bioethics is that we have the luxury of not falling into this same trap.

Finally, we have also defended the utility of speculative bioethics content even if there is no anticipated translational benefit. To avoid a future where bioethics is impoverished in its sources of inspiration, analogy, and examination or argument for its own sake, we must preserve the place of speculative bioethics.
